# A Case of Delusional Parasitosis With Folie à Deux Treated With Low-Dose Quetiapine

**DOI:** 10.7759/cureus.25344

**Published:** 2022-05-26

**Authors:** Christina Kalovidouri, Lukasz Kowalewski, Dragos Virgil Mos, Muhammad Umer Waqar

**Affiliations:** 1 Psychiatry, West London NHS Trust, Crowthorne, GBR; 2 Psychiatry, Royal College of Surgeons in Ireland, Dublin, IRL; 3 Psychiatry, Universitatea din Oradea, Oradea, ROU

**Keywords:** induced delusional disorder, delusional disorder, delusional infestation, ekbom syndrome, shared delusional infestation, folie à deux, delusional parasitosis

## Abstract

A patient with a three-month history of persistent delusions of infestation presented to the emergency department with suicidal ideation secondary to complaints of worsening pruritus. Routine investigations failed to disclose any underlying organic cause for her pruritic sensations. The patient ascribed these to a parasitic infestation acquired following a brief stay at her maternal aunt’s residence. Following a thorough psychiatric assessment and collateral history obtained from her aunt, it became clear that both the patient and her aunt held similar delusions of infestation. Her aunt was found to be the main inducer. The patient was admitted, successfully treated with low-dose quetiapine, and eventually deemed fit for discharge. Delusional parasitosis and folie à deux are both rare conditions that may sometimes co-occur.

## Introduction

Delusional parasitosis is a psychocutaneous disorder characterized by a resolute conviction that there is a parasitic infestation of the skin, despite the absence of any corroborating medical evidence. It is known by various names, such as Ekbom syndrome and delusional infestation. Patients will usually approach primary care physicians and dermatologists to seek treatment. An estimated 85% of dermatologists may encounter a case of delusional parasitosis at some point in their career [1]. Most patients will not be amenable to a referral for a psychiatric assessment, and neuroleptic medication may need to be encouraged by another physician after building a therapeutic rapport [2].

Delusional parasitosis can be broadly categorized as primary, secondary, and organic. In primary delusional parasitosis, the delusion of infestation is present but there are no concomitant psychiatric or organic conditions [3,4]. Secondary delusional parasitosis accompanies other mental disorders such as schizophrenia, depression, dementia, anxiety, and phobias [3]. The organic variant warrants the presence of an underlying medical condition such as anemia, hypothyroidism, diabetes, hepatitis, vitamin B12 deficiency, Parkinson’s and Huntington’s diseases, or a sexually transmitted infection such as HIV and syphilis [3]. It may also arise as a result of illicit drug use or due to prescribed medication [3,4].

Delusional parasitosis is more frequently encountered in middle-aged women and the average age of onset is usually around 57 years [5]. Female preponderance is noticeable in middle-aged patients but is not observed in younger patients [6]. Delusional parasitosis can be shared by more than one individual, usually among kinsfolk or members of the same household [6]. In these circumstances, the inducer typically persuades the other individual of their delusion, resulting in a shared delusional disorder [6]. Various studies report different frequencies of occurrence for this phenomenon, anywhere from 8% to 49% [6]. Successful management of the inducer is likely to result in the resolution of the condition in other individuals who had been affected [6].

## Case presentation

A 44-year-old woman self-presented to the emergency department complaining of pruritus and describing characteristic symptoms suggestive of epidermal parasitic skin diseases. She reported heightened levels of distress, complained of poor sleep, and expressed suicidal thoughts by stating that she wished to end her life by jumping in front of a moving vehicle or by ingesting insecticides. Routine laboratory investigations conducted were all within normal ranges. Electrocardiogram and urine toxicology screen were also clear. There was no objective evidence of cutaneous parasitosis. She was referred for a psychiatric evaluation.

She related that she had first noticed scalp pruritus approximately three months ago. At the time of presentation, she reported pruritic sensations in the axillary, submammary, pubic, intergluteal, popliteal, and umbilical regions as well as the interdigital spaces. Pruritus was reported as being worse at night. The patient recounted that she had initially approached her general practitioner, who had prescribed fexofenadine, permethrin, and benzyl benzoate. She had also taken ivermectin tablets, which she had purchased online. None of these therapies provided any symptomatic relief and, as a result, she developed suicidal ideation.

She was convinced that she had acquired this condition during a brief stay at the residence of her maternal aunt, who reportedly suffered from similar pruritic sensations. She believed that they both suffered from pediculosis and that her aunt’s home was “infested with parasites”. She provided a plastic container containing wipes and tapes with the remains of common household insects. She had discovered the remains of these in her surroundings and believed that they abided in her skin.

The patient had relocated to Dublin eight days ago from another Irish city. She was previously employed as an allied health professional in a teaching hospital elsewhere and now intended to move closer to her siblings. Since her arrival, she had been residing in her brother’s vacant accommodation. She had been sleeping on the floor to avoid infecting the mattresses and had cut her hair short to prevent parasites from residing therein. She was unwilling to visit her family and she did not want to take up employment for fear of spreading the parasitic infestation.

She was not known to any psychiatric service and she did not have any underlying medical conditions. She had undergone a myomectomy two years ago for uterine fibroids. She did not have any known allergies. She was an occasional consumer of alcohol and denied any illicit substance misuse. She also reported that she had previously quit smoking but had now resumed it on account of the “stress caused by parasites”. She was neither in a relationship nor sexually active. She described herself as a “perfectionist” who used to enjoy working overtime without any salary.

Mental status examination showcased a well-kempt woman in weather-appropriate attire, who had cropped hair and visible excoriations on her hands. She was interacting appropriately but would scratch her shoulders, legs, and hands from time to time. Her speech was relevant and coherent of normal rate, tone, and volume. Her mood was objectively euthymic, subjectively “low”, and her affect was reactive. She had tactile hallucinations, delusion of infestation, expressed suicidal thoughts, thoughts of hopelessness, and partial insight.

The patient consented for her maternal aunt to be contacted via telephone. Her aunt was 67 years old and resided alone in another Irish city, where the patient had also been residing previously. She recalled noticing tiny insects in her home over a year ago. Initially, she reported feeling “a stinging sensation” on her legs at night. She had injured her back several years ago and she suffered from sciatica. She attributed the tactile sensations to these at first but then stated that it progressed to a generalized sensation of pruritus and “crawling lice”. She suspected that she had scabies and approached her general practitioner, who ruled that out. Nevertheless, she continued to uphold the belief that she suffered from a hitherto unknown form of pediculosis. She added that she had tried various shampoos, ointments, and creams but found them all ineffective. She also complained that these insects had infested her nasal cavity and, on occasion, caused epistaxis. She cleaned her house regularly using pesticides to keep these insects at bay and did not allow anyone inside her dwelling. She confirmed that her niece had stayed in her house last year and believed that she too suffered from the same condition. She declined to engage with any psychiatric service and remained adamant that it would prove counterproductive.

The patient was offered a voluntary admission to a psychiatric ward. She accepted this reluctantly since she was hoping for admission to a medical ward. After admission, she reported feeling safe and denied any ongoing suicidal ideation. The delusion of infestation remained intact and she worried that she might cause a parasitic outbreak in the ward.

There was no evidence of any cutaneous disorder upon dermatological examination. Computed tomography (CT) head scan was also normal. The patient collected skin flakes from her bed and asked for these to be examined. The sample was sent to an entomology laboratory and no parasites were detected. The patient felt reassured by this outcome and eventually consented to trial quetiapine, which was initially commenced at 25 mg/day and then increased to 50 mg/day. She reported an improvement in her symptoms, her quality of sleep, and a reduction in distress. She no longer experienced any tactile hallucinations. She exhibited improved insight in terms of her ability to challenge her delusional experiences and her willingness to engage in medication management. She was subsequently discharged on quetiapine modified-release 100 mg/day after a duration of six weeks in the hospital. She remained concordant with her prescribed medication, less fixated on her delusion, and showed better self-care when reviewed at her follow-up outpatient appointment in the community.

## Discussion

The exact pathophysiological mechanisms underlying delusional parasitosis remain unclear and understudied. It has been hypothesized that deteriorated functioning of the striatal dopamine transporter, which also corresponds to an increased extracellular dopamine level, could potentially be an important etiological factor for delusional parasitosis [7]. Consequently, it is postulated that antipsychotics tend to ameliorate symptoms of delusional parasitosis by decreasing the hyperactive dopamine transmission [3].

In half of all cases, the source of infection is reported as being another person, especially individuals who return after sojourning in a developing nation [6]. Other perceived sources of infection include pets and previous experiences with pests, such as bed bugs and fleas, or parasitic cutaneous infections that have been addressed in the past [6]. The fictitious parasites are mostly believed to inhabit the skin, but may also be reported as residing in the nasal or oral cavities, genitalia, fecal matter, and in the immediate surroundings [6]. Specimens of these are collected in boxes, bottles, plastic bags, on adhesive tapes, and presented to a clinician for further examination. This behavior has been termed variously as “matchbox sign”, “Ziploc bag sign”, or “specimen sign” and is reported to occur in up to 70% of cases [3,6].

Two quintessential clinical symptoms characterize delusional infestation: an unshakeable belief, present for more than a month, that a macroscopic or microscopic organism has colonized the patient’s skin and the concomitant presence of tactile hallucinations, which patients often describe by using terms such as itching, crawling, burning, and tingling [3,4]. Aside from the delusion, patients are not detached from reality and cognitive capabilities remain intact [4]. Additional symptomatology may include specimen collection, social isolation, insomnia, folie à deux, anxiety, depression, and suicidal ideation [4]. These were all noted in the patient’s case presented previously. In addition, the patient’s maternal aunt evinced features of both orificial delusional infestation and delusory cleptoparasitosis, which are considered to be clinical variants of delusional parasitosis [8].

Dermatological examination of the skin fails to uncover any primary cutaneous lesions in patients with delusional infestation. Secondary cutaneous lesions that may be encountered include excoriations, lichenification, erosions, ulcers with hematic crusts, and scars [4,6]. These are the result of attempts, using mechanical force or chemical substances, to remove parasites and/or alleviate pruritus [3,6].

All other causes need to be excluded when delusional parasitosis is suspected and considering a broad differential is always essential. Cutaneous disorders such as xerosis, scabies, pediculosis capitis and pubis, eczema, prurigo, dermatitis herpetiformis, and an adverse reaction to medication should always be ruled out [6]. Other psychiatric diagnoses should also be taken into consideration; these could include anxiety disorders, phobias, and obsessive-compulsive disorder [9]. Organic causes should likewise be excluded. Recently, a case of right frontal meningioma masquerading as delusional parasitosis has also been reported [10].

Descriptions of shared delusional disorder and clinical subtypes thereof feature in literature as far back as the second half of the nineteenth century [11]. Folie à deux is further classified into four types: folie imposée, folie simultanée, folie communiquée, and folie induite [11]. The clinical presentation determines the subtype and, therefore, the therapeutic regimen needs to be tailored accordingly. In some cases, separation of secondary cases from the inducer may also be warranted as part of therapy [11,12]. Folie communiquée is the demonstrable subtype in this particular case based on the prolonged period of time it took for similar symptoms to manifest in the secondary case after the index case and their persistence despite geographical separation. Shared delusional infestation should always be considered as a possibility when conducting an initial assessment of patients presenting with delusional parasitosis [11].

Currently, insufficient high-quality data exist to support any evidence-based treatment for delusional disorders, including delusional parasitosis [13], hence it is recommended to include treatments effective for other psychotic disorders. Atypical antipsychotics are considered first-line therapies in most studies [3,4]. As per the critical literature review carried out by Freudenmann and Lepping, risperidone and olanzapine were found to be the most commonly used atypical antipsychotics [14]. Others support employing risperidone and aripiprazole as first-line and alternate first-line agents, respectively, whereas olanzapine and pimozide are relegated as second-line agents [15]. Escitalopram has also been successfully used to treat delusional parasitosis in some case reports [16].

There are four studies wherein quetiapine has been utilized for treating delusional parasitosis in a total of nine patients with dosages ranging from 25 to 800 mg/day [17]. An improvement in the patients’ symptoms was reported in all these studies [17]. In the case reported herein, low-dose quetiapine proved effective in treating this condition. Despite the favorable outcome, further studies and clinical trials are warranted before any veritable conclusions can be drawn. There are also studies suggesting that quetiapine is an inefficacious remedy for primary delusional parasitosis [4]. In one case report, quetiapine monotherapy at high doses failed to fully resolve the disorder until augmented with escitalopram [18]. Quetiapine is known to act as a hypnotic at doses lower than 300 mg/day and chiefly possesses antihistaminic properties [19]. Quetiapine’s antipsychotic action, similar to other atypical antipsychotics, stems not only from binding to Dopamine D2 receptors but also from 5-HT2A receptor blockade and partial agonistic activity at 5-HT1A receptors [19]. All these are insufficient at lower doses for a substantial antipsychotic response [19].

Differential diagnoses that can be considered in the case under discussion are somatization syndrome, obsessive-compulsive disorder, psychotic depression, and anxiety disorders. While the overall clinical picture lends credence to a diagnosis of induced delusional disorder, the symptomatic remission achieved with low-dose quetiapine allows room for the aforementioned diagnoses to be given due consideration. The form of delusional parasitosis is likely to have been secondary given that primary cases do not respond favorably to quetiapine [4]. In addition, underlying anxiety may also be a discernible feature in secondary cases of folie communiquée [11]. A thorough personality assessment might have yielded useful supplementary information as the patient in this scenario displayed anankastic personality characteristics and delusional infestation is more frequently encountered in persons with anankastic and paranoid traits [8].

## Conclusions

Delusional parasitosis, like other delusional disorders, is deemed a challenging disorder to treat by healthcare providers. It is exceedingly difficult to discuss a psychiatric referral with patients who present with this condition. Patients are often lost to follow-up once they become convinced that they are not being taken seriously by their treating physician. Liaison consultations involving dermatologists, entomologists, and psychiatrists would prove beneficial in the management of patients and prevention of any deterioration in mental state and self-inflicted cutaneous damage. It is hoped that further research will provide a better understanding of this disorder and possibly result in a standardized therapeutic approach to effectively address the needs of patients.
